# A Label-Free Luminescent Switch-On Assay for ATP Using a G-Quadruplex-Selective Iridium(III) Complex

**DOI:** 10.1371/journal.pone.0077021

**Published:** 2013-10-25

**Authors:** Ka-Ho Leung, Lihua Lu, Modi Wang, Tsun-Yin Mak, Daniel Shiu-Hin Chan, Fung-Kit Tang, Chung-Hang Leung, Hiu-Yee Kwan, Zhiling Yu, Dik-Lung Ma

**Affiliations:** 1 Department of Chemistry, Hong Kong Baptist University, Kowloon Tong, Hong Kong, China; 2 State Key Laboratory of Quality Research in Chinese Medicine, Institute of Chinese Medical Sciences, University of Macau, Macao, China; 3 Center for Cancer and Inflammation Research, School of Chinese Medicine, Hong Kong Baptist University, Hong Kong, China; Queen’s University Belfast, United Kingdom

## Abstract

We report herein the G-quadruplex-selective property of a luminescent cyclometallated iridium(III) complex for the detection of adenosine-5′-triphosphate (ATP) in aqueous solution. The ATP-binding aptamer was employed as the ATP recognition unit, while the iridium(III) complex was used to monitor the formation of the G-quadruplex structure induced by ATP. The sensitivity and fold enhancement of the assay were higher than those of the previously reported assay using the organic dye crystal violet as a fluorescent probe. This label-free luminescent switch-on assay exhibits high sensitivity and selectivity towards ATP with a limit of detection of 2.5 µM.

## Introduction

Adenosine-5′-triphosphate (ATP) plays a fundamental role in the normal physiological function of living organisms as the energy carrier of the cell [1]. It also involved in a variety of cellular metabolic and biochemical pathways. However, an abnormal concentration of physiological ATP has been implicated in the development of various diseases, such as angiocardiopathy [2]. Traditional analytical techniques for monitoring ATP concentration include mass spectrometry [3], enzyme-linked immunosorbent assays (ELISA) [4] and capillary electrophoresis (CE) [5]. However, these methods typically require expensive instrumentation and/or tedious sample preparation. As a consequence, it is desirable to develop more simple and rapid methods for the quantification of ATP.

Aptamers are short functional DNA or RNA oligonucleotides, produced *via* Systematic Evolution of Ligands by EXponential enrichment (SELEX), that bind to target molecules with high affinity and selectivity [6]. Some aptamers undergo conformational changes upon binding to the target molecules, allowing an appropriate DNA-interacting element to transduce the binding event into a luminescent, colorimetric, or electrochemical signal [7], [8], [9], [10], [11], [12]. The binding affinity of the ATP aptamer towards ATP has been described previously [13]. Using the ATP aptamer, a variety of oligonucleotide-based ATP detection platforms with luminescent [7], [14], [15], [16], [17], [18], [19], [20], [21], [22], colorimetric [23], [24], or electrochemical [25], [26], [27] outputs have been reported.

We have previously reported an ATP detection platform utilizing the organic dye crystal violet (CV) to monitor the ATP-induced G-quadruplex [14]. However, the sensitivity of the assay was limited somewhat by the promiscuous binding of CV to multiple DNA conformations. Meanwhile, transition metal complexes have emerged as attractive candidates for G-quadruplex-sensing applications due to the following reasons: (i) the long lifetime of ^3^MLCT phosphorescence enhances image signal stability and reduces background fluorescence noise, (ii) metal complexes can be made by simple synthetic protocols, (iii) the photophysical properties of metal complexes can be tuned by adjustment of the auxiliary ligands, and (iv) the relatively large stokes shifts helps to prevent self-quenching [28], [29], [30], [31], [32], [33], [34], [35], [36], [37], [38]. Encouraged by the previous application of G-quadruplex-selective metal complexes in DNA-based sensing platforms [13], [39], [40], [41], [42], [43], we sought to improve the sensitivity of our previous ATP detection assay by employing an iridium(III) complex as a G-quadruplex probe.

## Materials and Methods

### Materials

Reagents were purchased from Sigma Aldrich and used as received. Iridium chloride hydrate (IrCl_3_.xH_2_O) was purchased from Precious Metals Online. All reagents were used without further purification. Milli-Q purified water was used to prepare all solutions. All oligonucleotides were synthesized by Techdragon Inc. (Hong Kong, China). The sequences of the nucleic acid used in the study are display in table 1. The TRAMPC1 (ATCC® CRL2730™) cell line were purchased from American Type Culture Collection (Manassas, VA 20108 USA).

**Table 1:** The sequences of the nucleic acid used in the study.

ATP aptamer	5′-AACCTGGGGGAGTATTGCGGAGGAAGGT-3′
ATP aptamer complementary strand	5′-ACCTTCCTCCGCAATACTCCCCCAGGTT-3′
ATP aptamer mutant	5′-AACCTG*TTT*GAGTATTGCGGAG*T*AAG*T*T-3′
ATP aptamer mutant complementary strand	5′-A*A*CTT*T*CTCCGCAATACTC*AAA*CAGGTT-3′
HTS	5′-TTAGGGTTAGGGTTAGGGTTAGGG-3′
H21	5′-GGGTTAGGGTTAGGG TTAGGG-3′
ssDNA	5′- GAAATTCTTAAGTGCGATCGAG -3′

### General Experimental

Mass spectrometry was performed at the Mass Spectroscopy Unit at the Department of Chemistry, Hong Kong Baptist University, Hong Kong (China). Elemental analysis was performed at Atlantic Microlab Inc. (Norcross, GA, USA).

Deuterated solvents for NMR purposes were obtained from Armar and used as received. ^1^H and ^13^C NMR were recorded on a Bruker Avance 400 spectrometer operating at 400 MHz (^1^H) and 100 MHz (^13^C). ^1^H and ^13^C chemical shifts were referenced internally to solvent shift (CD_3_CN:^ 1^H, δ1.94, ^13^C, δ118.7; d_6_-DMSO: ^1^H, δ2.50, ^13^C δ39.5). Chemical shifts (δ) are quoted in ppm, the downfield direction being defined as positive. Uncertainties in chemical shifts are typically ±0.01 ppm for ^1^H and ±0.05 for ^13^C. Coupling constants are typically ±0.1 Hz for ^1^H-^1^H and ±0.5 Hz for ^1^H-^13^C couplings. The following abbreviations are used for convenience in reporting the multiplicity of NMR resonances: s, singlet; d, doublet; t, triplet; q, quartet; m, multiplet; br, broad. All NMR data was acquired and processed using standard Bruker software (Topspin).

Absorption spectra were recorded on a Cary 300 UV/Vis spectrometer. Emission spectra were recorded on a PTI QM4 spectrometer. Quantum yields and lifetime measurements were performed on a PTI TimeMaster C720 Spectrometer (Nitrogen laser: pulse output 337 nm) fitted with a 380 nm filter. Error limits were estimated: λ (±1 nm); τ (±10%); 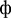 (±10%). All solvents used for the quantum yield and lifetime measurements were degassed using three cycles of freeze-vac-thaw.

Luminescence emission spectra were recorded on a PTI QM-4 spectrofluorometer (Photo Technology International, Birmingham, NJ) at 25°C, with the slits for both excitation and emission set at 2.5 nm. The sample cell was a 0.7 mL quartz cuvette. The luminescence intensity at 550–750 nm was monitored after excitation of the sample at 360 nm with a xenon lamp excitation source, using 90° angle detection for the solution samples.

### Synthesis of [Ir(ppy)_2_(biq)]PF_6_ (1) and [Ir(piq)_2_(biq)]PF_6_ (2)

The following compounds were prepared using literature methods: [Ir_2_(ppy)_4_Cl_2_], [Ir_2_(piq)_4_Cl_2_] [44] and [Ir(ppy)_2_(biq)]PF_6_ (**1**), [Ir(piq)_2_(biq)]PF_6_ **(2)** [44] and characterized by ^1^H-NMR,^13^C-NMR and HRMS.

### Emission Response of 2 Towards Different Forms of DNA

The G-quadruplex DNA-forming sequences (HTS and H21) were annealed by incubating at 95°C for 10 min, allowed to cool to room temperature at 0.1°C/s in Tris-HCl buffer (25 mM Tris, 50 mM KCl, pH 7.0) and stored at –20°C before use. **2** (1 µM) was added to 5 µM of ss DNA, ct DNA or G-quadruplex DNA in 500 µL of Tris-HCl buffer (25 mM Tris, pH 7.0). Emission spectra were recorded in 550−700 nm range using an excitation wavelength of 360 nm.

### G-quadruplex Fluorescent Intercalator Displacement (G4-FID Assay)

The FID assay was performed as previously described [42]. The ATP aptamer G-quadruplex (0.25 µM) in Tris-HCl buffer (50 mM Tris, 100 mM KCl, pH 7.0) was annealed by heating at 95°C for 10 min. Thiazole orange (0.5 µM) was added and the mixture was incubated for 1 h. Emission measurements were recorded after each addition of the indicated concentrations of complexes, following an equilibration time of 5 min after each addition. The fluorescence area was converted into percentage of displacement (PD) by using the following equation. PD = 100−[(FA/FA_0_)×100] (FA_0_ = fluorescence area of DNA-TO complex in the absence of complexes; FA = fluorescence area in the presence of complexes).

### Complex 2 as a G-quadruplex Probe for Oligonucleotide-based ATP Detection

12.5 µL of 100 µM ATP aptamer and its complementary DNA was mixed in hybridization buffer (50 µL, 20 mM Tris-HCl, pH 7.3). The mixture was annealed at 90°C for 10 min, and was slowly cooled down from 90°C to 25°C. This stock solution of 25 µM duplex DNA was stored at –20°C for further use. In the emission measurement, the duplex strand stock solution was diluted with Tris-HCl buffer (20 mM, pH 7.3) to obtain a 0.25 µM (in final volume 500 µl) aptamer duplex solution in a cuvette. Various concentrations of ATP (final concentration ranging from 0 to 10 mM) were added to each cuvette, followed by the addition 1 µl of 500 µM of the G-quadruplex probe at a final concentration of 1 µM. The mixture was allowed to equilibrate at 25°C for 10 min.

### Total Cell Extract Preparation

The TRAMPC1 (ATCC® CRL2730™) cell line were purchased from American Type Culture Collection (Manassas, VA 20108 USA). Prostate cancer cells were trypsinized and resuspended in TE buffer (10 mM Tris-HCl 7.4, 1 mM EDTA). After incubation on ice for 10 min, the lysate was centrifuged and the supernatant was collected. The cell extract was then spiked with ATP and the luminescence spectra were recorded after the addition of **2** (1 µM) and DNA duplex (0.25 µM) and equilibration at 25°C for 10 min.

## Results and Discussion

**Figure 1:**
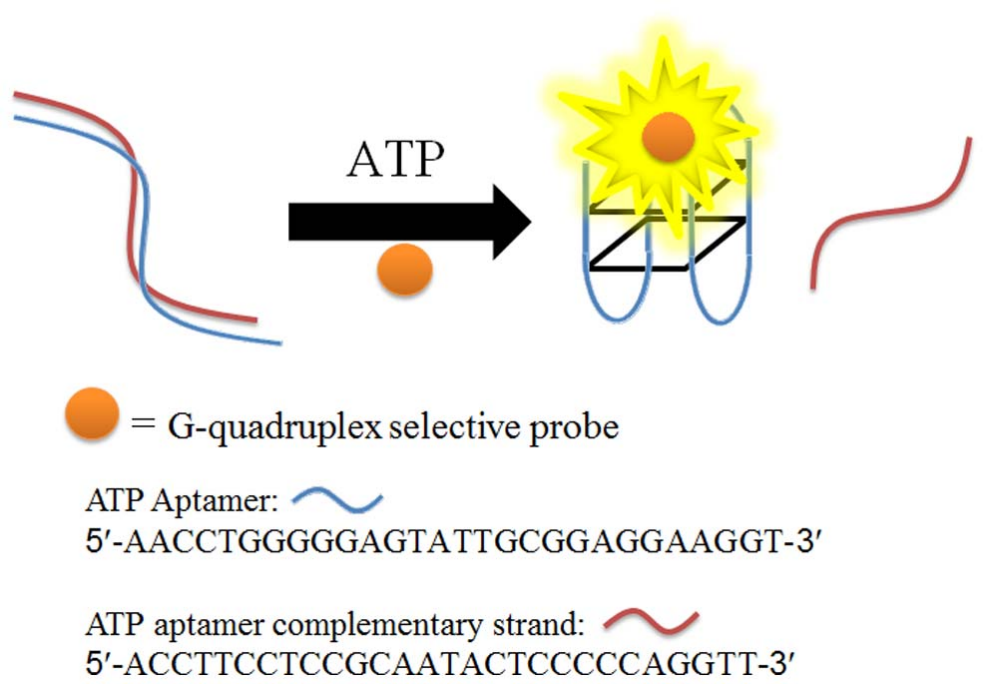
Schematic illustration of the G-quadruplex-based assay for the detection of ATP.

The mechanism of this ATP sensing platform is depicted in Figure 1. Initially, the ATP aptamer hybridizes with its complementary sequence to form a double-stranded DNA (dsDNA) structure. In the absence of ATP, DNA duplex interacts weakly with the G-quadruplex-selective iridium(III) complex, resulting in a low emission signal. The addition of ATP induces the dissociation of the duplex structure *via* the formation of an aptamer-target complex, in which the ATP aptamer adopts a G-quadruplex structure. The strong interaction of the iridium(III) complex with the G-quadruplex motif results in an enhanced luminescence response.

**Figure 2:**
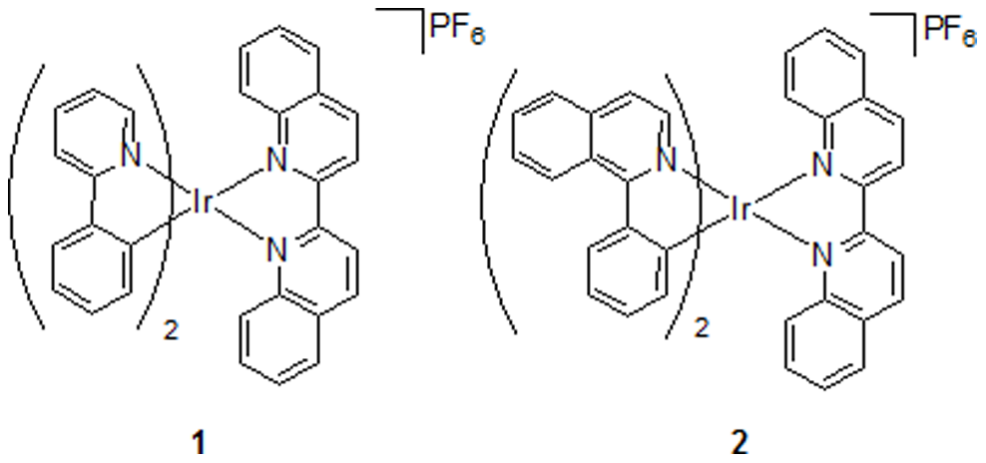
Chemical structure of cyclometallated iridium(III) complexes 1 and 2.

**Figure 3:**
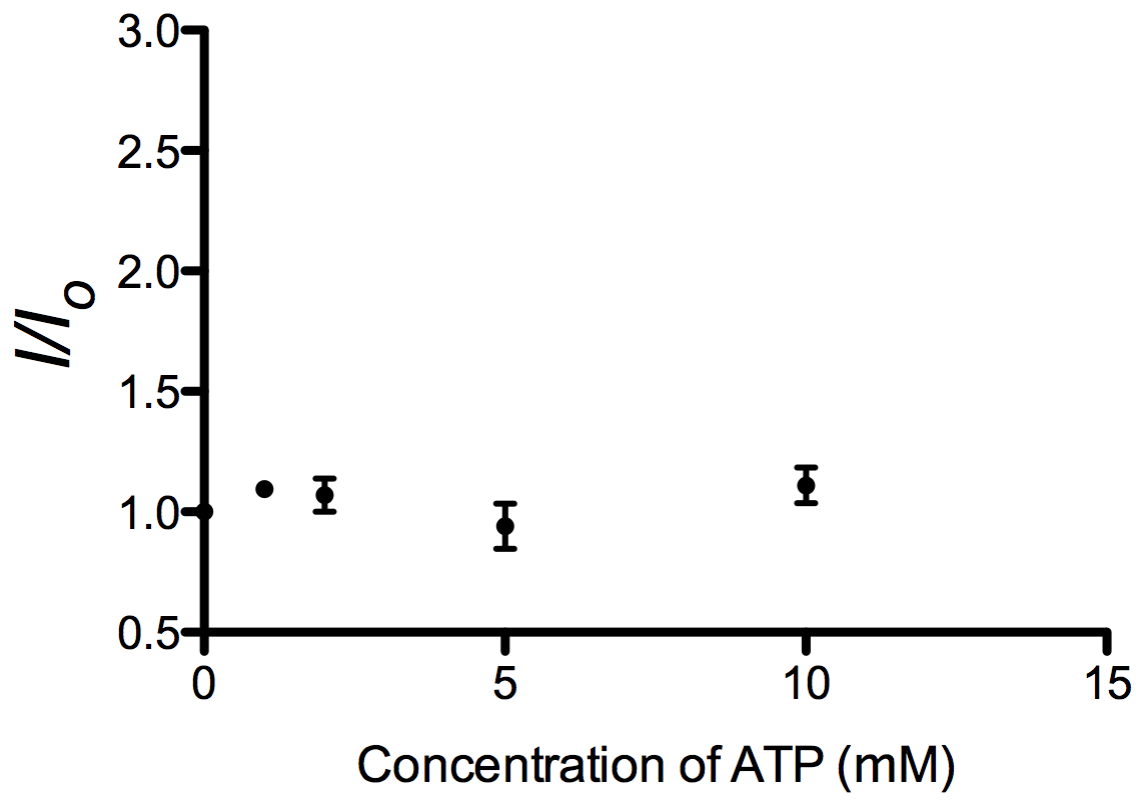
Emission spectrum of the 1/duplex system in response to various concentrations of ATP: 0, 1, 2, 5 and 10 mM. λ_ex_ = 360 nm.

To evaluate the feasibility of this strategy, we first investigated the luminescence intensity of the system in response to different concentrations of ATP, and utilizing the previously reported G-quadruplex-selective cyclometallated iridium(III) complex **1** [Ir(ppy)_2_(biq)]PF_6_ (where ppy = 2-phenylpyridine, biq = 2,2′-biquinoline) (Figure 2) as the signal transducer. Surprisingly, no significant increase of luminescence intensity was observed even in the presence of 10 mM of ATP (Figure 3). The low luminescence enhancement of this system was presumably due to the weak interaction between **1** and the ATP aptamer G-quadruplex, as the complex has been reported to only bind to only certain types of G-quadruplex structures [45]. We anticipated that by increasing the size of the ligands, the resulting complex might form superior π-stacking interactions with the G-quadruplex tetrads or loops. Therefore, we investigated the application of complex **2** [Ir(piq)_2_(biq)]PF_6_ (where piq = 1-phenylisoquinoline) (Figure 2) containing two planar extended ligands in the label-free G-quadruplex-based ATP sensing platform.

**Figure 4:**
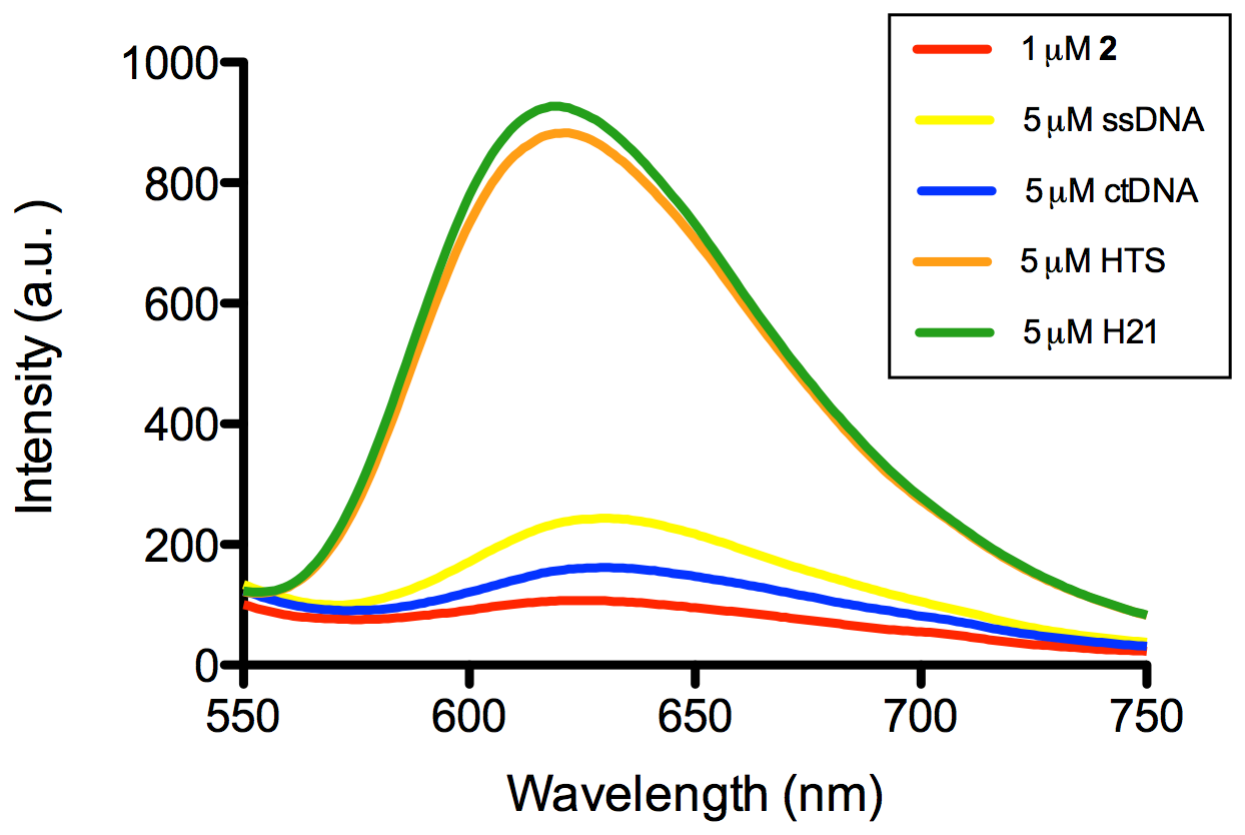
Luminescence response of complex 2 (1 µM) in the presence of 5 µM of single stranded DNA (ssDNA), calf-thymus DNA (ctDNA) or various G-quadruplexes (HTS, H21). λ_ex_ = 360 nm.

The synthesis and characterization of the cyclometallated iridium(III) complex **2** was performed according to a previous report [44]. We investigated the luminescence response of complex **2** towards various types of DNA, including single-stranded DNA (ssDNA), double-stranded calf-thymus DNA (ctDNA) and pre-annealed G-quadruplex DNA (Figure 4). Complex **2** was weakly emissive in aqueous buffered solution (20 mM Tris, 50 mM KCl, pH 7.0). Encouragingly, the presence of G-quadruplex DNA sequences (HTS, H21) could significantly enhance the luminescence of complex **2**. A maximum 8-fold enhancement was observed in the luminescence intensity of **2** at 5 µM of the H21 and HTS G-quadruplex (Figure 4).

**Figure 5:**
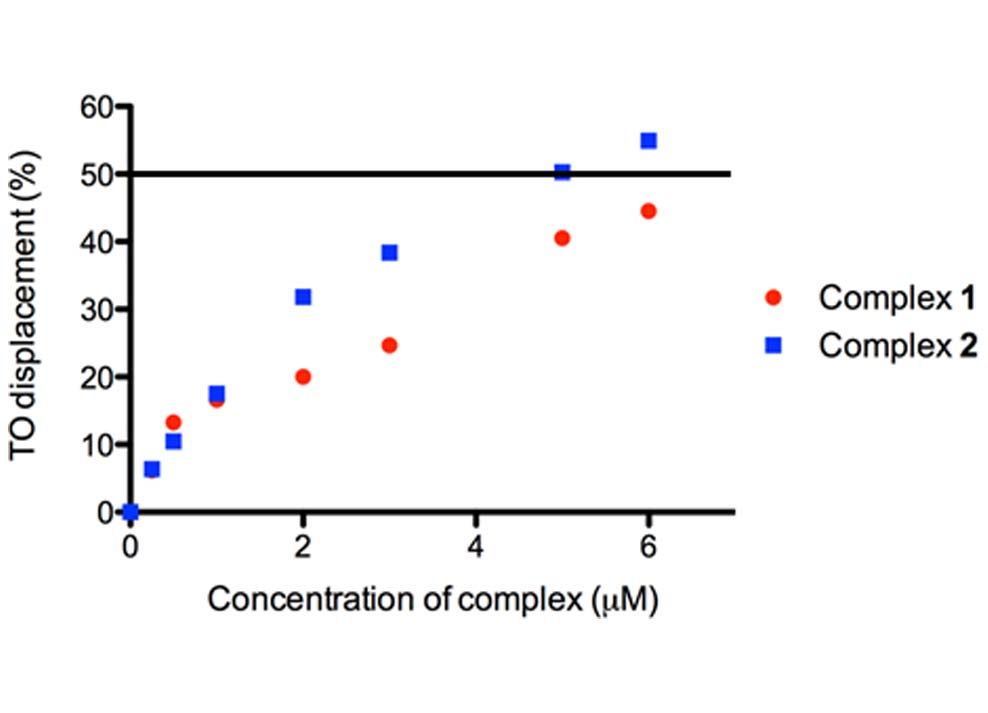
G4-FID titration curves of the ATP G-quadruplex in the presence of increasing concentration of complex 1 and 2 in Tris-HCl buffer (50 mM Tris, pH 7.0, 100 mM KCl). DC_50_ value is determined by half-maximal concentration of compound required to displace 50% TO from DNA.

To investigate the binding affinity of complex **1** and **2** towards the pre-annealed ATP aptamer G-quadruplex, the G-quadruplex fluorescent intercalator displacement (G4-FID) assay was employed. The results of the G4-FID assay indicated that **2** could displace thiazole orange (TO) from the ATP aptamer G-quadruplex with a ^G4^DC_50_ value (half-maximal concentration of compound required to displace 50% TO from DNA) of 5 µM, while the ^G4^DC_50_ value of **1** was >6 µM (Figure 5). This result indicates that complex **2** has higher binding affinity towards the ATP aptamer G-quadruplex compared to complex **1**. We envisage that the strong interaction between complex **2** and the G-quadruplex protects the metal center from the aqueous buffer environment and suppresses non-radiative decay, thus enhancing ^3^MLCT emission. By comparison, the addition of ssDNA or ctDNA resulted in only minimal changes in the luminescence of complex **2**. To our knowledge, complex **2** has not previously been reported as a G-quadruplex-selective probe.

**Figure 6:**
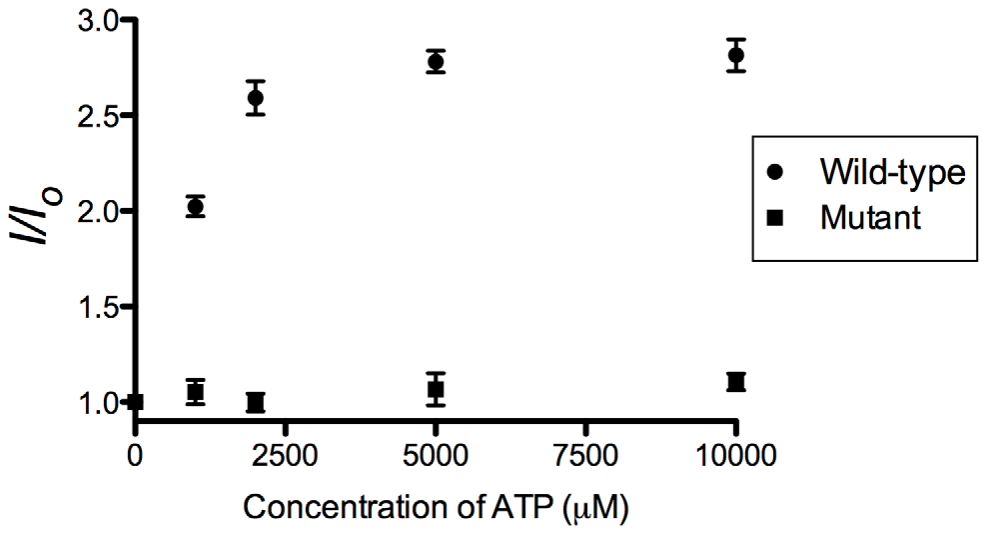
Emission spectrum of the 2/duplex (wild-type/mutant) system in response to various concentrations of ATP: 0, 1, 2, 5 and 10 mM. λ_ex_ = 360 nm.

Based on established selective luminescence response of complex **2** towards G-quadruplex DNA, we sought to employ complex **2** in the oligonucleotide-based ATP detection assay. In the absence of ATP, complex **2** was only slightly emissive due to the weak interaction between the metal complex with the duplex substrate (Figure 6). Encouragingly, the luminescence signal of **2** was significantly enhanced in the presence of ATP, presumably due to the formation of the aptamer G-quadruplex induced by ATP.

**Figure 7:**
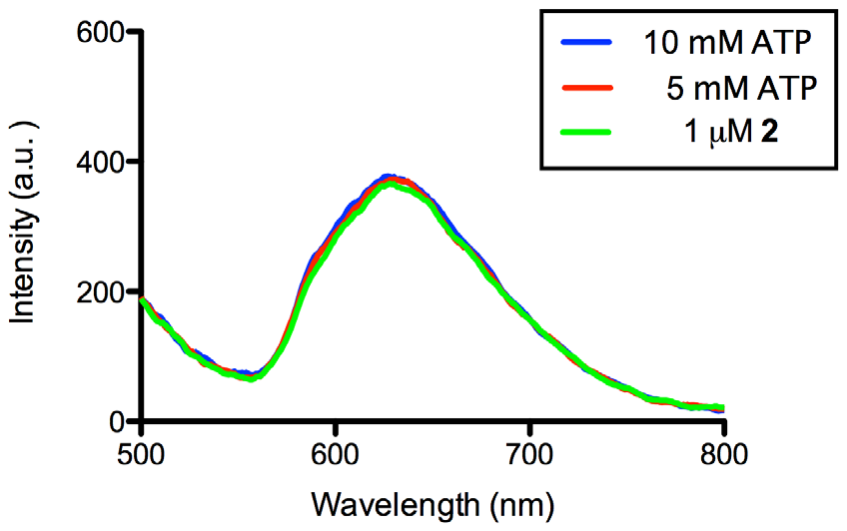
Emission spectrum of the 2 upon addition of 5 and 10λ_ex_ = 360 nm.

A number of control experiments were performed to validate the mechanism of the proposed assay. We investigated the response of a modified system involving a mutant sequence (5′-AACCTG*TTT*GAGTATTGCGGAG*T*AAG*T*T-3′, base mutants underlined) that cannot form a G-quadruplex due to the lack of critical guanine residues (Figure 6). No luminescence enhancement was observed upon addition of 1, 2, 5 of 10 mM of ATP to the modified system. This suggests that the complex **2** specifically interacts with the G-quadruplex motif formed after the addition of ATP, rather than through non-specific DNA-ATP-**2** ternary interactions. We have previously demonstrated using circular dichroism (CD) spectroscopy that ATP could induce the formation of the G-quadruplex structure from a dsDNA substrate containing the ATP aptamer sequence [14]. Furthermore, incubating complex **2** with 5 or 10 mM of ATP resulted in no significant luminescent enhancement, indicating that the metal complex does not directly interact with ATP (Figure 7).

**Figure 8:**
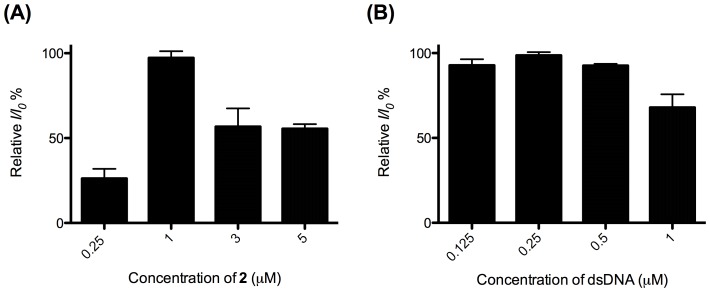
Relative luminescence intensity of the system (A) ([dsDNA] = 2 µM, [ATP] = 5 mM) in the presence of different concentrations of complex 2 (0.25, 1, 3 and 5 µM) in aqueous buffered solution (50 mM Tris, pH 7.2). (B) ([complex 2] = 1 µM, [ATP] = 5 mM) at various concentrations of the dsDNA (0.25, 0.5, 1.5 and 3 µM) in aqueous buffered solution (50 mM Tris, pH 7.2). Error bars represent the standard deviations of the results from three independent experiments.

**Figure 9:**
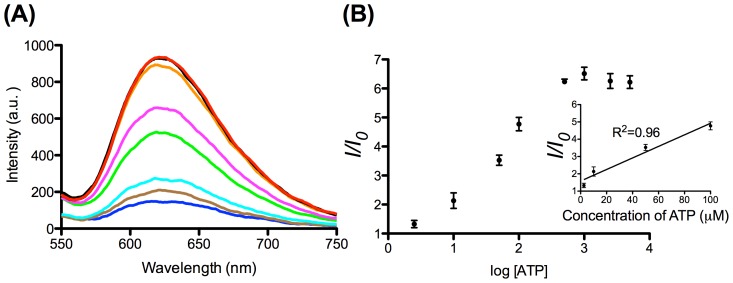
Emission spectrum of the 2/duplex system ([**2**] = 1 µM, [dsDNA] = 0.25 µM) in the presence of increasing concentrations of ATP (0, 2.5, 10, 50, 100, 500, 1000, 2500 and 5000 µM). λ_ex_ = 360 nm (left). Luminescence response of the system vs. log [ATP]. Inset: linear plot of the change in luminescence intensity of the system vs. ATP concentration (right).

In order to optimize the luminescence response of this sensing platform, we investigated the effect of the concentration of dsDNA and complex **2**. We observed that the luminescence intensity in the presence of 5 mM ATP attained a maximum intensity when the concentrations of dsDNA and complex **2** were 0.25 µM and 1 µM, respectively (Figures 8A and B). The luminescence response of this platform increased with ATP concentration and reached saturation at 1 mM of ATP in buffered solution (Figure 9A), with a linear (*R* ^2^ = 0.96) luminescence response in the range of 2.5−100 µM of ATP (Figure 9B) Under the optimized conditions, the maximal fold-change enhancement was improved to 6.5-fold compared to *ca.* 2.5-fold for the unoptimized system (Figure 6). Furthermore, the limit of detection (LoD) recorded using the 3σ method was 2.5 µM. The performance of the present system is superior to that in our previous study using the organic dye CV (fold change = 4, LoD = 5 µM). We envisage that the superior sensitivity of the present system could be attributed to the highly selective interaction between iridium(III) complex **2** and G-quadruplex DNA. In contrast, CV also interacts significantly with duplex DNA, resulting in a higher fluorescent background signal leading to lower sensitivity and maximal fold-change response.

**Figure 10:**
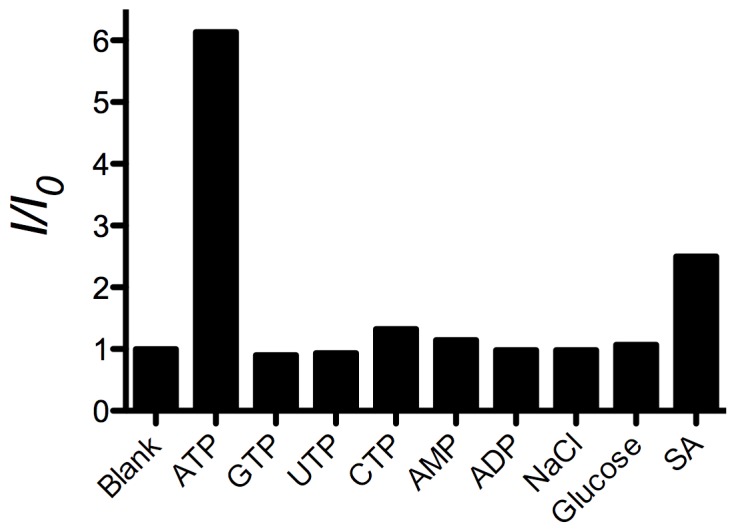
Luminescence fold change of the system for 1′-triphosphate (UTP), guanosine 5′-triphosphate (GTP), cytidine 5′-triphosphate (CTP), adenosine 5′-diphosphate (ADP), adenosine 5′-monophosphate (AMP), 300 mM NaCl, 2% glucose and 0.05% serum albumin (SA).

**Figure 11:**
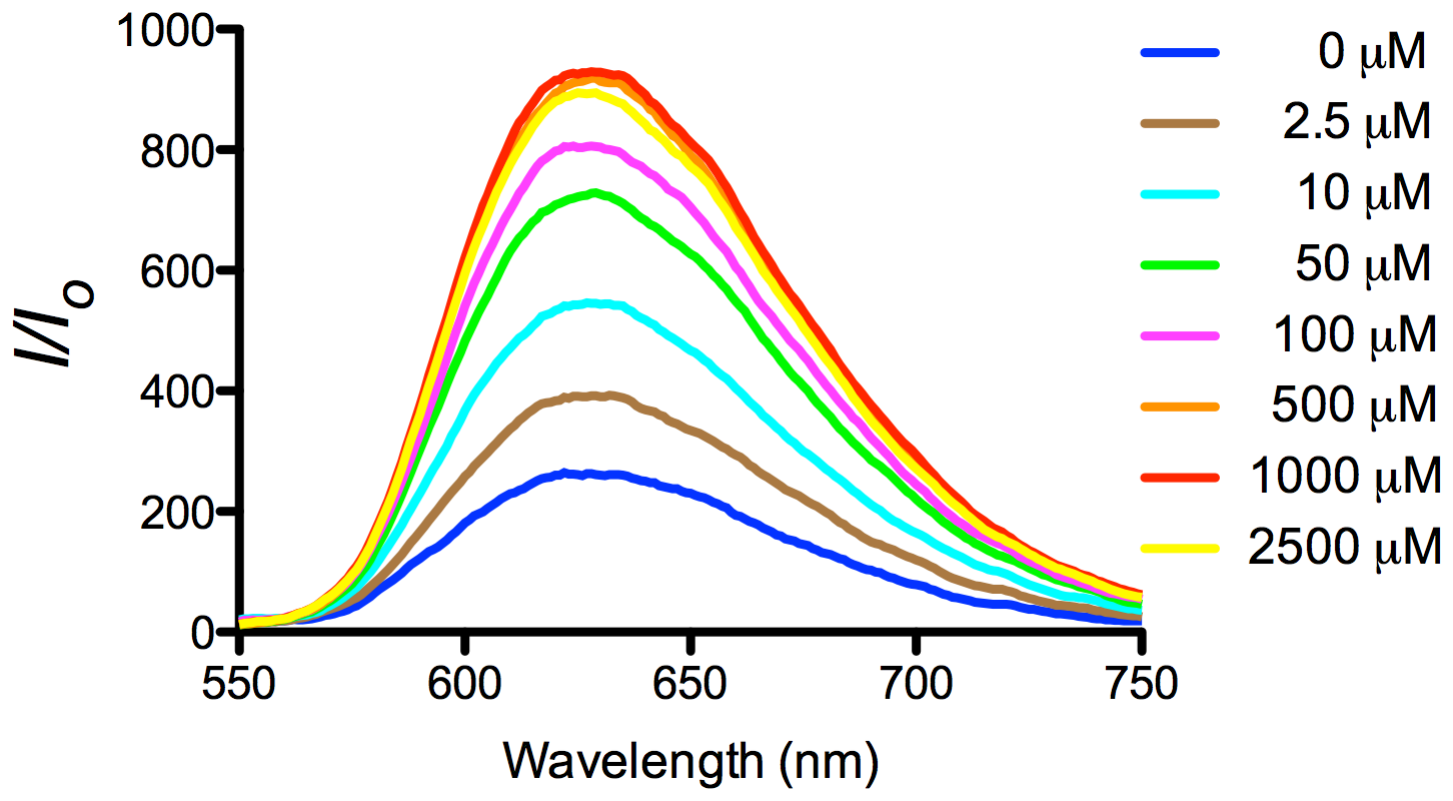
Emission spectrum of the 2/duplex system ([2] = 1 µM, [dsDNA] = 0.25 µM) in the presence of increasing concentrations of ATP (0, 2.5, 10, 50, 100, 500, 1000 and 2500 µM) in whole cell extracts. λ_ex_ = 360 nm.

The selectivity of this platform towards ATP over related analogues such as uridine 5′-triphosphate (UTP), guanosine 5′-triphosphate (GTP), cytidine 5′-triphosphate (CTP), adenosine 5′-diphosphate (ADP) and adenosine 5′-monophosphate (AMP) was also investigated. While a high luminescence signal was recorded in the presence of 1 mM ATP, only small changes in the luminescence intensity of the system was observed in the presence of 10-fold excess amounts of the ATP analogues (Figure 10). Furthermore, we also investigated the selectivity of this ATP detection platform towards other species that may be present in biological samples, such as NaCl, glucose, and serum albumin. The assay did not generate significant luminescence signal in the presence of NaCl (300 mM) or glucose (2%). On the other hand, 0.05% of serum albumin induced an emission enhancement that was 25% that observed with 1 mM of ATP. Overall, these results demonstrate the selectivity of this platform towards ATP. In order to evaluate the robustness of the system, we investigated the ability of the assay to detect ATP in spiked total cell extracts. Encouragingly, the emission signal of the system was enhanced upon increasing concentrations of ATP (Figure 11). This result demonstrates the potential application of the ATP detection platform for real sample analysis.

## Conclusions

In conclusion, a G-quadruplex-based luminescent iridium(III) switch-on assay has been developed for the selective detection of ATP in aqueous solution. Our sensing platform is label-free, rapid, sensitive, simple, cost-effective, and displays a switch-on response with detection limit 2.5 µM. Furthermore, we demonstrated the potential application of this strategy for the detection of ATP in biological samples. This platform is based on the ATP aptamer and the heretofore unreported G-quadruplex-selective property of complex **2**, which exhibit a strong luminescence in the presence of the ATP-induced G-quadruplex. On the other hand, the original iridium(III) complex **1** was found not to generate luminescence enhancement with the ATP aptamer G-quadruplex, though it had previously been reported to recognize other types of G-quadruplexes. This study highlights the importance of structural modification on the selectivity of luminescent iridium(III) complexes for various G-quadruplex topologies. The exact relationship between iridium(III) complex structure and G-quadruplex selectivity is still under investigation.
